# 3D imaging of the human temporal bone by X-ray phase-contrast tomography

**DOI:** 10.1038/s44303-025-00086-y

**Published:** 2025-05-20

**Authors:** Jannis J. Schaeper, Paul Tafforeau, Christoph A. Kampshoff, Carolina Thomas, Alexander Meyer, Christine Stadelmann, M. Charles Liberman, Tobias Moser, Tim Salditt

**Affiliations:** 1 Institute for X-ray Physics, University of Göttingen, Göttingen, Germany; 2 Cluster of Excellence “Multiscale Bioimaging: From Molecular Machines to Networks of Excitable Cells”, University of Göttingen, Göttingen, Germany; 3 Beamline BM18, European Synchrotron Radiation Facility, Grenoble, France; 4 Institute for Auditory Neuroscience, University Medical Center Göttingen, Göttingen, Germany; 5 Department of Otolaryngology, University Medical Center Göttingen, Göttingen, Germany; 6 Institute for Neuropathology, University Medical Center Göttingen, Göttingen, Germany; 7 Eaton-Peabody Laboratories, Massachusetts Eye and Ear Infirmary, Boston, MA USA; 8 Department of Otolaryngology, Head and Neck Surgery, Harvard Medical School, Boston, MA USA

**Keywords:** Biophysics, Medical imaging, Imaging techniques

## Abstract

Studying the subtle and intricate three-dimensional structure of the human cochlea embedded in the temporal bone requires structure-preserving imaging approaches with adaptable field of view and resolution. Synchrotron X-ray phase-contrast tomography at the novel beamline BM18 (EBS, ESRF) offers the unique capability to achieve histological resolution at the scale of the entire organ, based on high lateral coherence, long propagation distances, and optimized spectral range. At the same time advances in laboratory *μ*-CT instrumentation and protocols also open up new opportunities for 3D micro-anatomy and histopathology, including 3D reconstruction of nerve tissue when suitable staining protocols are used. Here we report on post mortem 3D imaging of human temporal bones and excised human cochleae, both unstained and stained to visualize the auditory nerve. Further, we highlight the use of this imaging modality for development of novel cochlear implant technology.

## Introduction

Disabling hearing loss affects 5% of the world’s population. Access to the micro-anatomy of the ear is crucial for understanding sensorineural hearing loss and for the development of cochlear implants (CIs) for hearing restoration. The subtle and intricate three-dimensional (3D) structure of the cochlea encased in the temporal bone requires structure-preserving imaging approaches. The cochlea has been studied with 2D imaging approaches such as conventional histology^1^ and transmission electron microscopy^2^ which offer high 2D resolution, but are susceptible to slicing and staining artifacts. Furthermore, coverage of a large volume is extremely tedious. Light sheet fluorescent microscopy (LSFM)^3^ offers 3D imaging of the cochlea, but requires tissue clearing which takes time and is also associated with tissue shrinkage and structural changes. Magnetic resonance imaging (MRI)^4^ on the other hand is non-destructive, but does not resolve cells.

X-ray phase-contrast computed tomography (XPCT) allows for non-destructive imaging of the human temporal bone or excised cochlea and is compatible with different tissue preparation techniques such as staining with osmium tetroxide (OTO) which increases contrast for lipid-rich tissue, dehydration in an increasing ethanol series to increase contrast between tissue and solvent and even tissue clearing allowing multi-modal imaging with LSFM^3^. There are a number of XPCT-studies on rodent and primate animal models used in auditory research both in unstained^3,5–8^ and OTO- or iodine-stained specimen^9–12^. In the translation from rodent to non-human primate to human cochleae, the sample size increases and thus requires a wider field-of-view (FOV), a higher photon energy, and eventually also adaptation of the phase-retrieval schemes. Human temporal bones have been studied in a number of XPCT-studies both at synchrotron (SR) sources, including both unstained and non-decalcified^1,13–18^, unstained and paraffin embedded^19^, as well as OTO stained samples^5,20^. Tackled questions include the evaluation of cochlear duct length and tonotopic mapping^14,15,21^ and analysis of the cochlear anatomy for therapeutic intervention^16,17,22^. Human cochleae have also been studied with μ-CT liquid embedded and unstained^13^, OTO- or iodine-stained^11,12,17^ and dried^23^.

Modern fourth generation synchrotron sources such as the Extremely Brilliant Source (EBS) at the European Synchrotron Radiation Facility (ESRF) offer highest brilliance and coherence even at comparatively high photon energies, allowing the imaging of large intact human organs with μm resolution. The *HiP-CT* protocol^24^ describes optimal sample preparation. In order to reduce sample movement and bubble formation and to increase contrast organs are transferred to 70% ethanol (EtOH) and mounted in agarose. At the test beamline BM05, brain, heart kidney, spleen and lung^25–27^ have been studied. The new beamline BM18^28^ has been explicitly designed to scan very large samples with a polychromatic, high-energy beam exploiting propagation-based phase-contrast. Recently hearts^29^ and kidneys^30^ have been scanned at BM18.

**Fig. 1:**
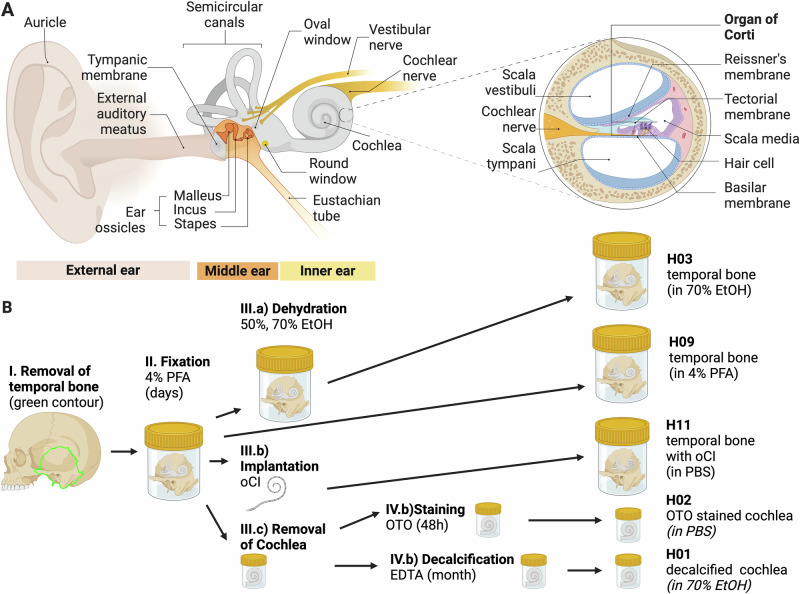
Schematic representation of the ear anatomy and sample preparation. **A** The 3D structure of the middle and inner ear, especially the organ of Corti (OoC) with the hair cells and their innervation by the spiral ganglion neurons are of interest for auditory research. **B** Sample preparation for XPCT experiments. Temporal bones were retrieved during routine autopsy and fixed in 4% paraformaldehyde (PFA) for several days. One temporal bone was kept in 4% PFA (H09). Another temporal bone was dehydrated in an increasing ethanol (EtOH) series up to 70% EtOH (H03). A third temporal bone was implanted with an optical cochlear implant (oCI) and kept in PBS. Two additional temporal bones got trimmed down to the cochlea of which one was decalcified and dehydrated to 70% EtOH (H01). The other cochlea was stained with osmium tetroxide (OTO) and kept in PBS (H02). All samples were mounted in cylindrical jars with agarose to avoid sample movement and degassed to avoid bubble formation. Fig. created with *Bio Render*.

In this paper we report on post-mortem 3D imaging of excised human temporal bones and human cochleae using highly brilliant synchrotron radiation at the beamline BM18, GINIX instrument at beamline P10 and in-house μ-CT. We explore different sample mountings in PFA, PBS and 70% EtOH, as well the OTO stain for better visibility of the nervous tissue, see Fig. 1 for an overview of the samples considered for this manuscript. Importantly, along with the imaging approaches described below and the discussion of the results, we include all 3D datasets as a benchmark, for further tests of segmentation and morphometric measurements and for use in modeling sound transduction or fitting of implants.

## Results

### Experiment design and image chain

**Fig. 2:**
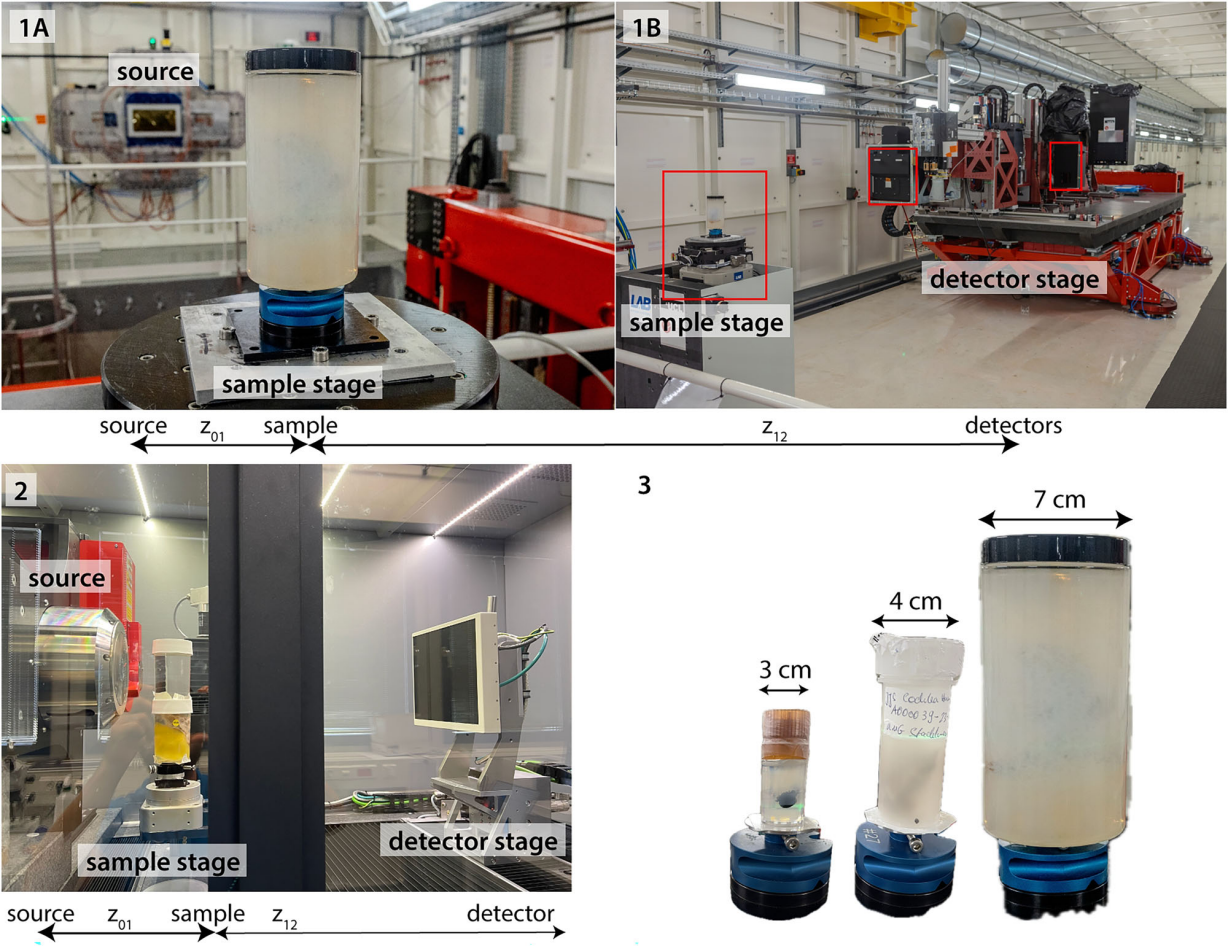
Synchrotron and *μ*-CT setups. (1) BM18 beamline, ESRF, showing (**A**) exit window and sample stage and (**B**) sample stage and detector stage with different detector-lens combinations. The flatfield images are usually taken in a sample-free region of the sample jar. (2) μ-CT setup with source, sample stage and detector. The flatfield images are taken in a control jar that is filled with the same solvent as the sample jar. (3) Sample jars used for different sample sizes such as the excised cochlea and the temporal bone.

While absorption contrast in X-ray CT is well suited to image dense materials such as bones and metal implants, contrast for soft tissue can be enhanced by exploiting the sample-induced phase shift of (partially) coherent X-rays. Phase shifts arise from the decrement *δ*(**r**) of the X-ray refractive index *n*(**r**) = 1 − *δ*(**r**) + *iβ*(**r**) which is orders of magnitude larger for biological soft tissue than the imaginary part *β*(**r**) related to absorption. The sample-induced phase-shifts are converted into measurable intensity modulations by free-space propagation. Depending on the effective propagation distance *z*_eff_ = *z*_12_/*M*, the effective pixelsize dx_eff_ = dx/*M* and the photon wavelength *λ*, the imaging regime can be described by the unitless Fresnel number $F={{\rm{dx}}}_{{\rm{eff}}}^{2}/({z}_{{\rm{eff}}}\lambda )$. Sample preparation is key for multiscale XPCT. Samples are partially dehydrated, kept in PBS or stained and mounted in cylindrical jars with agarose, see Fig. 1 for an overview of sample preparation and the Methods section for details. Sample containers as well as annotated photos of the BM18 beamline are shown in Fig. 2.

The BM18 beamline is located at the EBS storage ring. The detector stage houses several detector-lens combinations and can be translated to change the propagation distance *z*_12_. The BM18 source is a tripole wiggler with a fixed gap. The polychromatic energy *E* can be tuned between 50 and 280 keV by inserting polished attenuators. The polychromatic energy is carefully tailored in view of the object size, material composition, dose limit, and the dynamic range of the detector. Maintaining the dose limit is critical so that no bubbles are created which introduce streak artifacts and motion artifacts^31^. An effective energy *E*_eff_ can be calculated by taking into account the transmission of the entire optical pathway and detection parameters of the scintillator screen, exemplary calculated spectra are shown in the Supplementary Fig. 1.

**Fig. 3:**
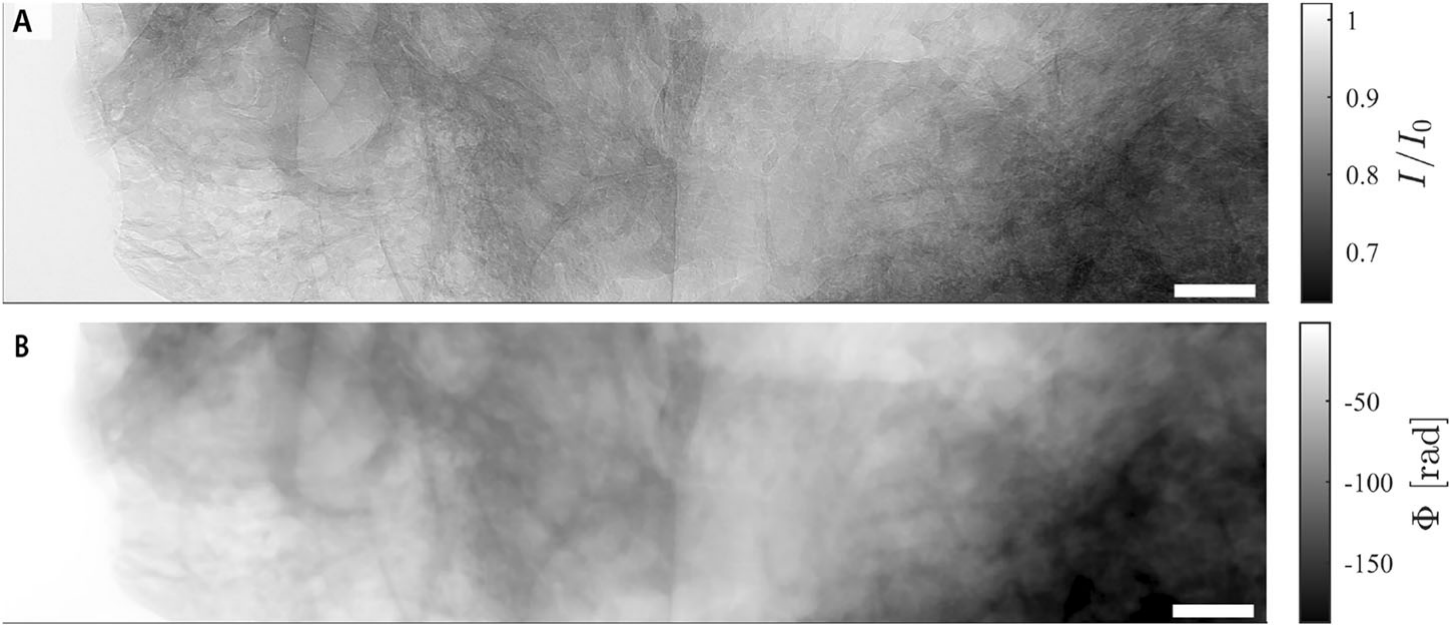
Typical XPCT projections from overview scans performed at BM18 with dx_eff_ = 6.2 μm (sample 11). **A** Flatfield-corrected projection image (sample H11) and **B** corresponding phase-reconstruction with Paganin scheme, *δ*/*β* = 1000, *F* = 0.7716. Scale bars 2 mm.

For the large temporal bones, overview scans were recorded at a voxelsize dx_eff_ = 6.2 μm in half-acquisition mode to almost double the detector’s horizontal FOV to 58 mm. To accommodate for the absorption gradient through the sample jar, the sample was shifted to the side of the beam profile. Flatfield images were recorded in the sample container in a sample-free region following the HiP-CT protocol^24^. Phase-reconstruction was performed with Paganin’s algorithm^32^. An illustrative projection image of the temporal bone and phase-retrieval are shown in Fig. 3. Region-of-interest (ROI) tomography of temporal bones and cochleae were performed with the rotation axis in the middle of the detector, placed in the middle of the symmetric beam profile at dx_eff_ ∈ {1.8, 2.3} μm. In all configurations, accumulation of images can be exploited to increase signal-to-noise ratio. The *μ*-CT setup is shown in Fig. 2 (2). Scan times were significantly longer. Adapting the accumulation time to the required detail- and noise-level typically resulted in overnight scans between 12 and 16 h. The propagation distance *z*_12_ and the magnification *M* were set by moving both sample and detector stage. The flatfield was recorded in a separate container filled with the same solvent and mounted above sample jar.

In the following we present datasets of (i) human temporal bone in 4% PFA (H09), (ii) human temporal bone post-mortem implanted with oCI in PBS (H11), (iii) OTO stained cochlea in PBS (H02) and (iv) decalcified Cochlea in 70% EtOH (sample H01). See Tables 1, 2, 3 and 4 in the Methods section for detailed information on samples and scan parameters for both the synchrotron setup and *μ*-CT setup.

**Table 1:** Overview of the samples presented in this manuscript

Parameter	H01	H02	H03	H09	H11
Description	Cochlea	Cochlea	Temporal bone	Temporal bone	Temporal bone
Decalcified	10% EDTA	/	/	/	/
Stain	/	OTO	/	/	/
Implant	/	/	/	/	oCI
Solvent	70% EtOH	PBS	70% EtOH	4% PFA	PBS
Container diameter (cm)	3	3	4	4	7

**Table 2:** Acquisition parameters for human cochleae (H02, H09, H11) measured at BM18

Parameter	H02 overview	H02 zoom	H09 overview	H09 Zoom	H11 overview
Energy *E*_eff_ (keV)	94	75	92	64	125
Filter Mo (mm)	/	/	/	/	1.3
Filter Al_2_O_3_ (mm)	5	5	5	/	/
Filter SiO_2_ (mm)	15	/	/	/	/
Filter glassy carbon (mm)	90	80	85	90	/
Detector	IRIS 15	PCO	IRIS 15	IRIS 15	IRIS 15
Objective	DZoomOptic	tandem Hasselblad (100/300)	DZoomOptic	ZoomOptic	DZoomOptic
Magnification *M*	0.626	3	0.626	4.661	0.626
Scintillator (μm)	LuAG:Ce 2000	LuAG:Ce 200	LuAG:Ce 2000	LuAG:Ce 100	LuAG:Ce 2000
Exposure time (ms)	5 × 25	3 × 45	3 × 25	3 × 35	5 × 15
Acquistion time (min)		11	38	42	136
Pixelsize dx (μm)	4.25	6.5	4.25	4.25	4.25
Binning	/	/	/	2-fold	/
Voxelsize dx_eff_ (*μ* m)	6.2	2.3	6.2	1.8	6.2
Sample-detector *z*_12_ (m)	5	2	5	1	5
Projections	4000	6000	10,000	8000	15,000
COR offset (px)	0	900	400	1000	2200
Tomograms	1	3	3	3	7
Volume size (mm^3^)	23 × 23 × 10	9 × 9 × 13	36 × 36 × 32	8 × 8 × 7	58 × 58 × 43
Volume size (number of pixels)	3659 × 3659 × 1601	3843 × 3843 × 5596	5897 × 5897 × 5233	4548 × 4548 × 4137	9449 × 9449 × 7024
Dataset size (GB)	40	154	340	159	1100

**Table 3:** Acquisition parameters for human cochleae (H01, H03) measured at BM18 and GINIX

Parameter	H01 – BM18	H01 – GINIX	H03 overview	H03 Zoom
Energy *E* mono (keV)	/	20	/	/
Energy *E*_eff_ (keV)	64	/	92	64
Filter glassy carbon (mm)	90	/	5	90
Detector	IRIS 15	PCO edge	IRIS 15	IRIS 15
Objective	ZoomOptic	10× zoom	DZoomOptic	ZoomOptic
Magnification *M*	4.661	/	0.626	4.661
Scintillator (μm)	LuAG 100	LuAg 50	LuAG 2000	LuAG 100
Exposure time (ms)	10 × 25	35	3 × 25	3 × 35
Binning	2	/	/	/
Pixelsize px (μm)	4.25	6.5	4.25	4.25
Voxelsize vx (μm)	1.81	0.65	6.2	1.8
Sample-detector *z*_12_ (m)	1	0.029	5	1
Projections	8000	3000	4000	8000
COR offset (px)	1000	0	0	1000
Tomograms (*z*)	4	1	1	3
Volume size (mm^3^)	8 × 8 × 9	1.3 × 1.3 × 0. 8^a^	32 × 32 × 12	8 × 8 × 7

^a^GINIX: per single tomogram, the cochlea was almost covered by 3 × 3 × 4 scans.

**Table 4:** Acquisition parameters for human cochleae *μ*-CT

Parameter	H02	H11 overview	H11 zoom
Source tube voltage (kV)	80	120	120
Source	Microfocus	Microfocus	Microfocus
Source spot mode	small	middle	small
Tube power (W)	10	30	10
Detector	Flat panel	Flat panel	Flat panel
Magnification *M*	17.64	4.23	8.17
Exposure time (s)	99 × 9.36	14 × 0.4	8 × 4
Acquisition times (h)	12	4.25	16
Voxelsize dx_eff_ (μm)	7.2	30	15.53
FOV (mm^2^)	10.4 × 12.3	52.1 × 36.4	28 × 28 × 21
*z*_01_ (mm)	35.4	78.6	78.6
*z*_02_ (mm)	623.0	332.9	642.9
Projections	1120	1440	1440

### Human temporal bone in fixative solution

**Fig. 4:**
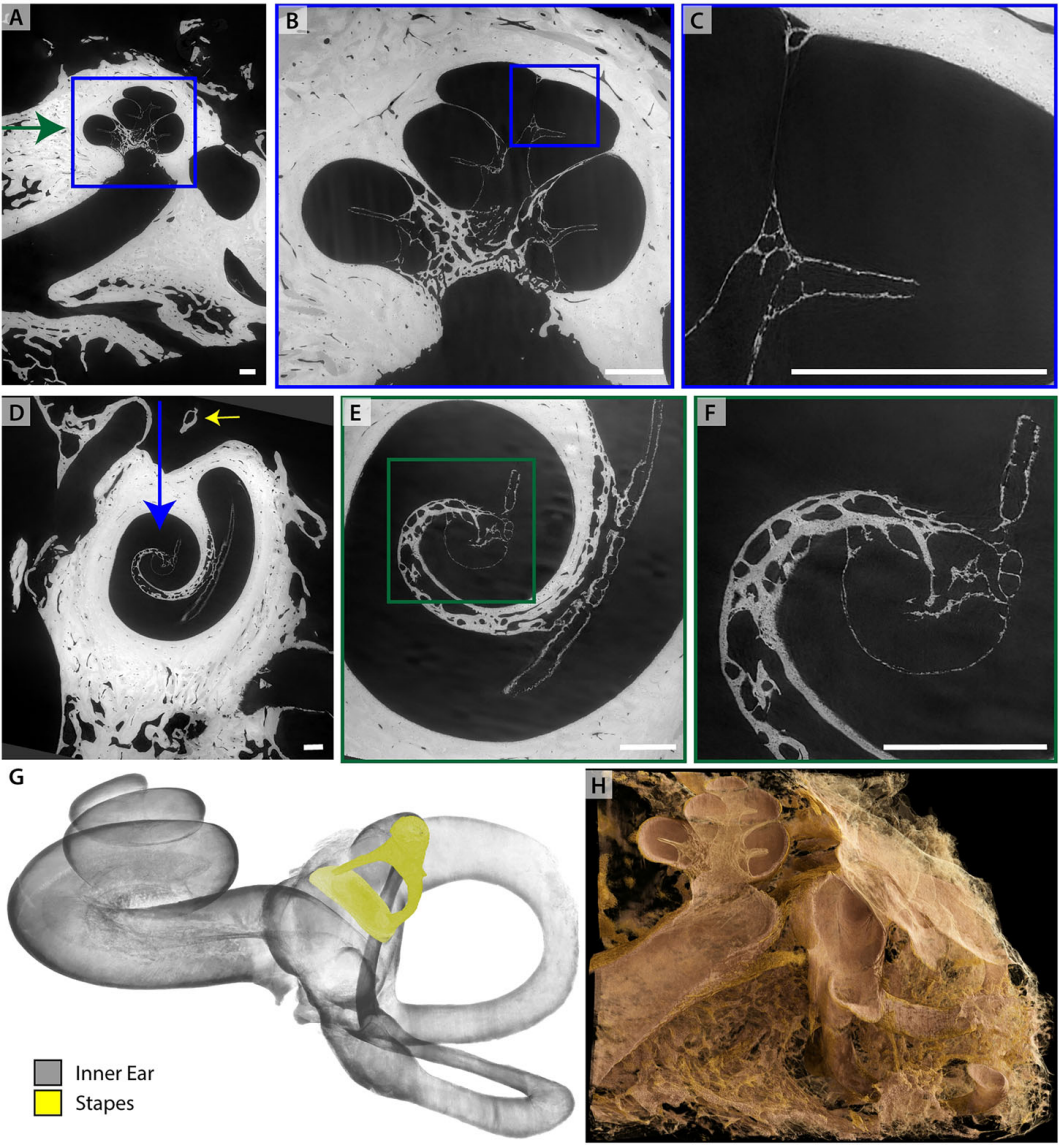
XPCT of human temporal bone in 4% PFA (sample H09) recorded at BM18. **A** Virtual slice through reconstruction volume at dx_eff_ = 6.2 μm. **B** ROI tomogram recorded at the position of the cochlea at dx_eff_ = 1.8 μm. **C** Zoom into (**B**). **D** Virtual slice through the reconstruction volume, perpendicular to (**A**). **E** ROI tomography at the position of cochlea. **F** Zoom into (**E**). **G** Volume rendering of the segmented cochlea and stapes. **H** Volume rendering of unsegmented temporal bone cut for unobstructed view. Scale bars 1 mm. (**A**–**C**) share the same orientation and are perpendicular to (**D**, **E**). The position of the respective perpendicular slices is indicated by arrows.

First, we showcase the multiscale imaging capability of BM18 for a human temporal bone immersed in 4% PFA and mounted in a jar of diameter 4 cm (sample H09). The overview scan was performed with dx_eff_ = 6.2 μm in a half acquisition scheme. Subsequently a ROI tomography at the position of the cochlea was recorded at dx_eff_ = 1.8 μm. Virtual slices through the reconstruction volume are shown in Fig. 4. The bony structures in the cochlea are represented well and at high detail level, see for example the fine structure in the surrounding bone. However, soft tissue such as the OoC is nearly invisible in this imaging configuration. As we show further below, the OoC becomes highly contrasted, however, when using heavy atom labeling (Fig. 7), and also when decalcified (Fig. 9). Note that (A-C) is oriented perpendicular to (D-F). The high contrast level and resolution facilitated fast segmentation based on region growing. Renderings of the segmented cochlea and stapes, as well as of the unsegmented temporal bone are shown in (G) and (H) respectively.

### Human temporal bone with oCI

**Fig. 5:**
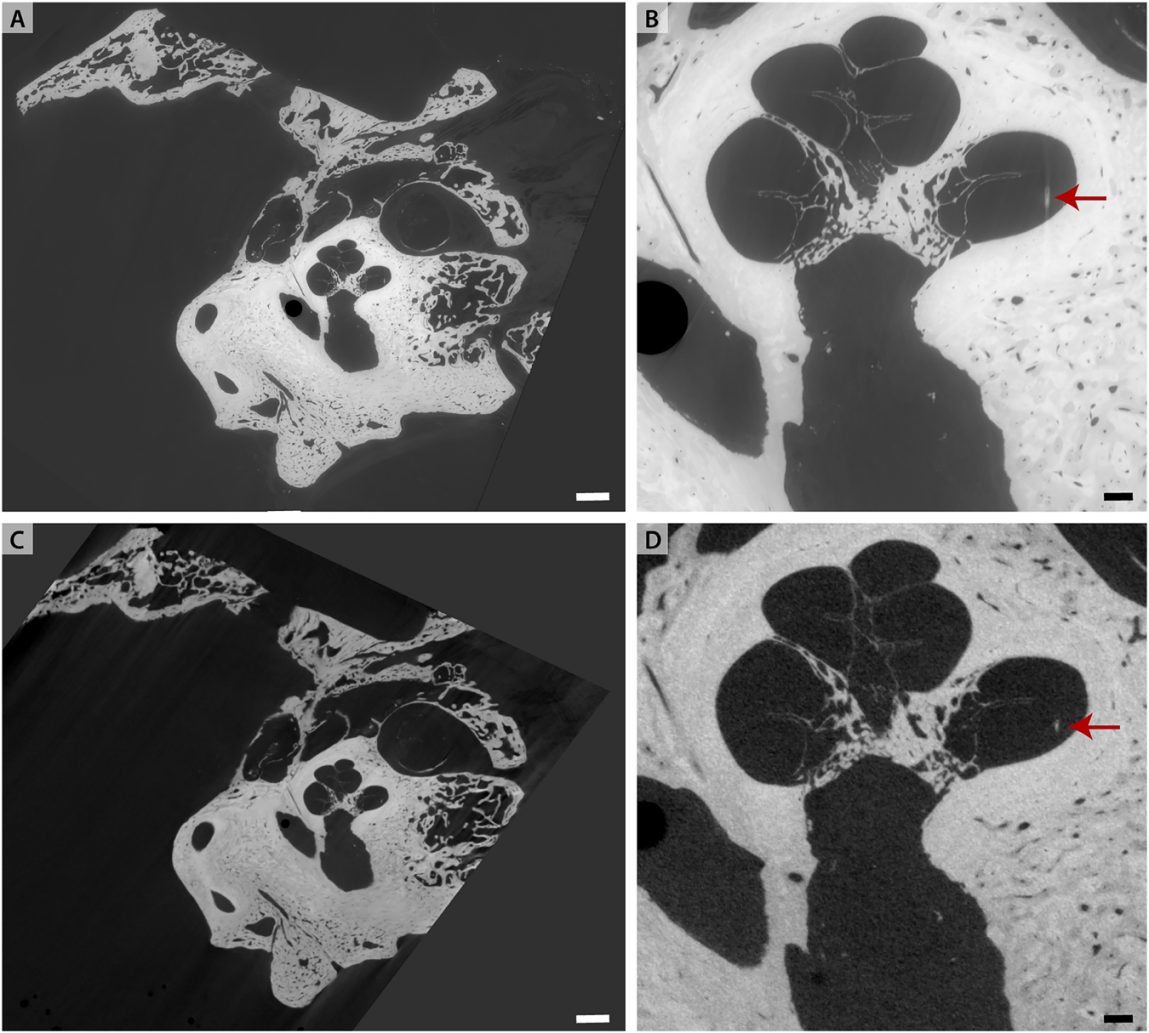
XPCT of human temporal bone (sample H11). **A** Overview scan recorded at BM18 with dx_eff_ = 6.2 μm, and shown here after 2-fold binning. **B** Zoom of (**A**). **C** *μ*-CT scan of the same sample, dx_eff_ = 30 μm. **D** Region-of-interest tomography of (**C**) with dx_eff_ = 15.6 μm. The red arrow points at the oCI. Scalebar left 2.5 mm, scalebar right 0.5 mm.

**Fig. 6:**
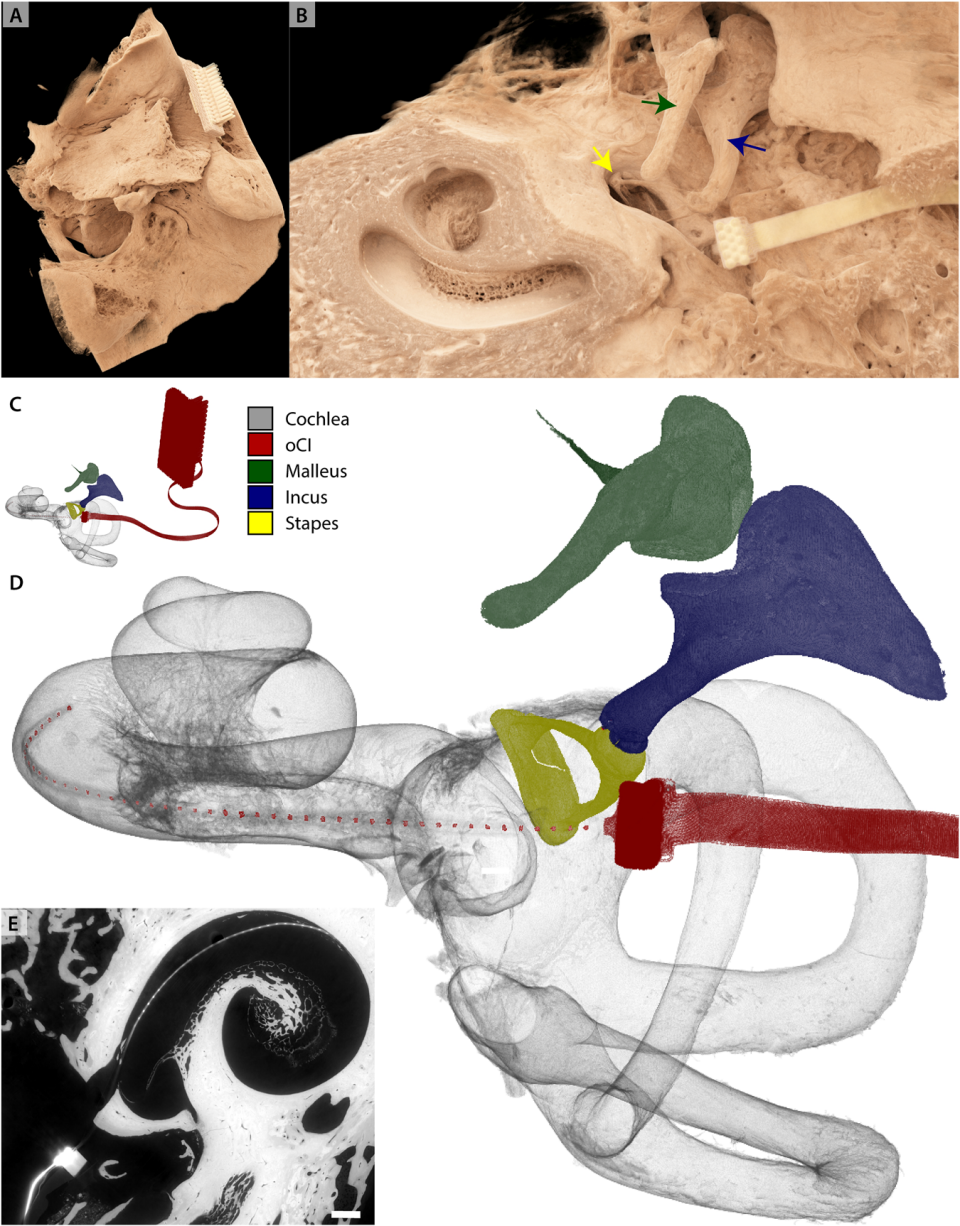
Volume rendering of human temporal bone implanted with oCI (sample H11) as introduced in Fig. 5 recorded at BM18. **A** Volume rendering of the whole dataset. **B** Zoom into the middle and inner ear, clipping part of the dataset for an unobstructed view in the middle ear. The three ossicles are marked with arrows. **C** Volume rendering of segmented ossicles, oCI and cochlea. **D** Zoom into (**B**). Note the placement of the oCI's LEDs in the basal turn of the cochlea. **E** Virtual slice through the reconstruction volume showing the placement of the oCI in the basal turn of the cochlea. Scale bar 1 mm. **A**–**D** share the same orientation. See Supplementary Movie 1 for an animation.

Next, we present datasets of a human temporal bone which had been implanted with an oCI post-mortem. The sample was immersed in PBS and mounted in a jar of diameter 7 cm (sample H11). The overview scan was recorded at dx_eff_ = 6.2 μm in a half acquisition scheme. The same sample was scanned additionally at the *μ*-CT-setup, at dx_eff_ = 30 μm and at dx_eff_ = 15.6 μm, respectively. Since the sample had to be remounted during beamtime, the reconstruction volumes of these three datasets have been aligned manually. Virtual slices through the reconstruction volume are shown in Fig. 5. The fine bony structures of the cochlea are fairly represented and a high detail level in the surrounding bone is visible. Note that even in the ROI *μ*-CT dataset most of the fine lines are all likely to be bony trabeculae rather than unmineralized soft tissue such as membranes. At the same time, the surrounding bone shows a high detail level, although the SNR for the *μ*-CT is significantly lower than at BM18. Furthermore, the oCI implant results in a small but visible beam hardening artifact (see arrow). The high contrast in the SR-dataset allowed fast segmentation and volume rendering. The oCI and the three ossicles malleus, incus and stapes have been segmented with a region growing algorithm. The volume rendering in Fig. 6 shows the placement of the oCI in the basal turn of the cochlea. Supplementary Movie 1 presents an animation of the temporal bone.

### OTO stained cochlea

**Fig. 7:**
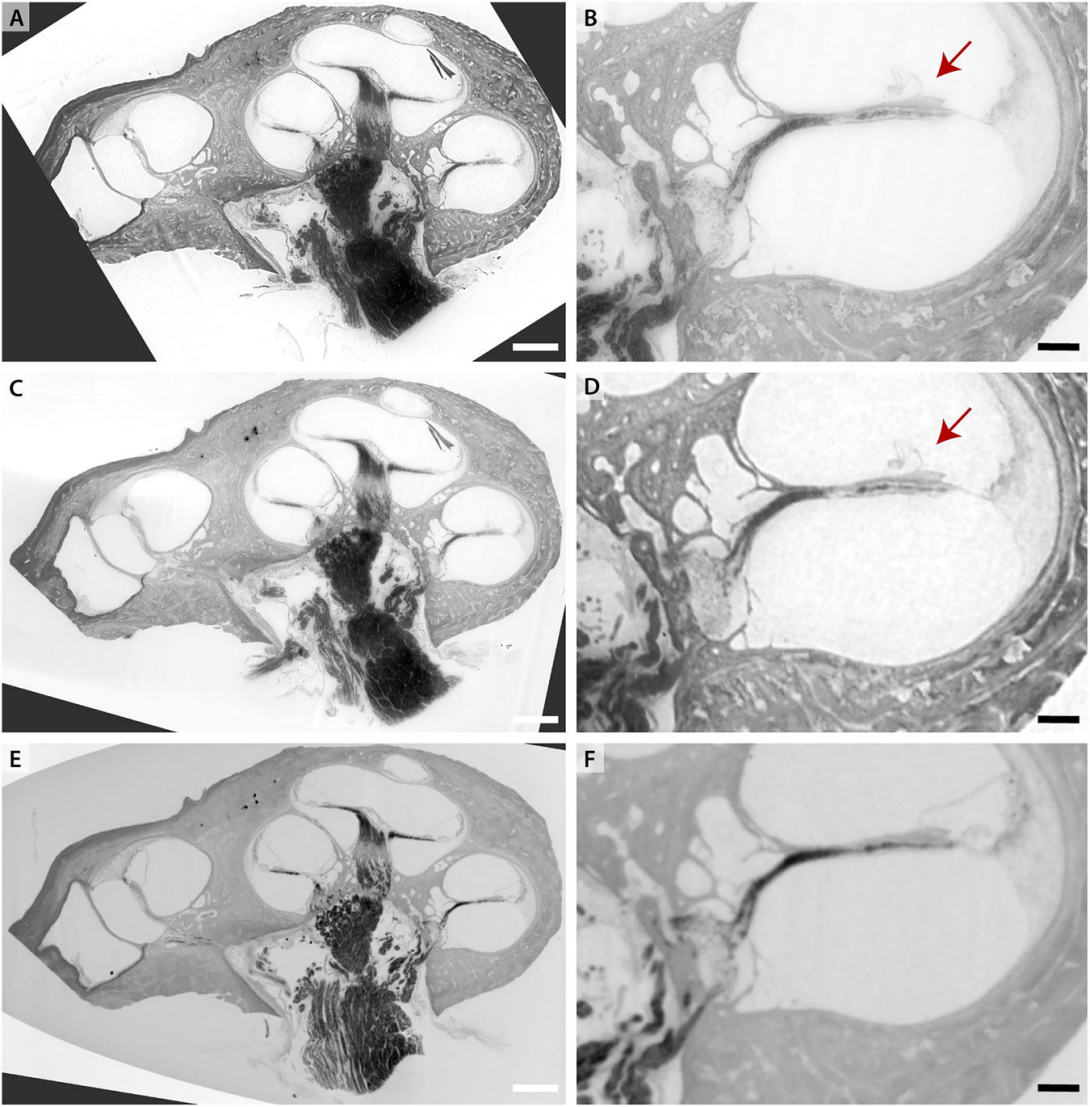
Imaging the OTO stained human cochlea (sample H02). The OTO stain increases contrast for the lipid-rich nervous tissue. The reconstructed volumes obtained by different instruments and settings have been aligned virtually. **A** BM18 with dx_eff_ = 2.3 μm. **B** Zoom into (**A**). **C** BM18 overview scan with dx_eff_ = 6.2 μm and (**D**) Zoom into (**C**). **E** *μ*-CT with dx_eff_ = 7.2 μm with (**F**) Zoom into (**E**). The red arrows point at Reissner’s membrane, which appears ruptured in the BM18 data, while it is intact in the prior recorded inhouse *μ*-CT scan. Note also that the gray values are inverted with respect to standard radiography images, i. e. dark corresponds to high density. Scale bars: (left) 1 mm, (right) 0.25 mm.

**Fig. 8:**
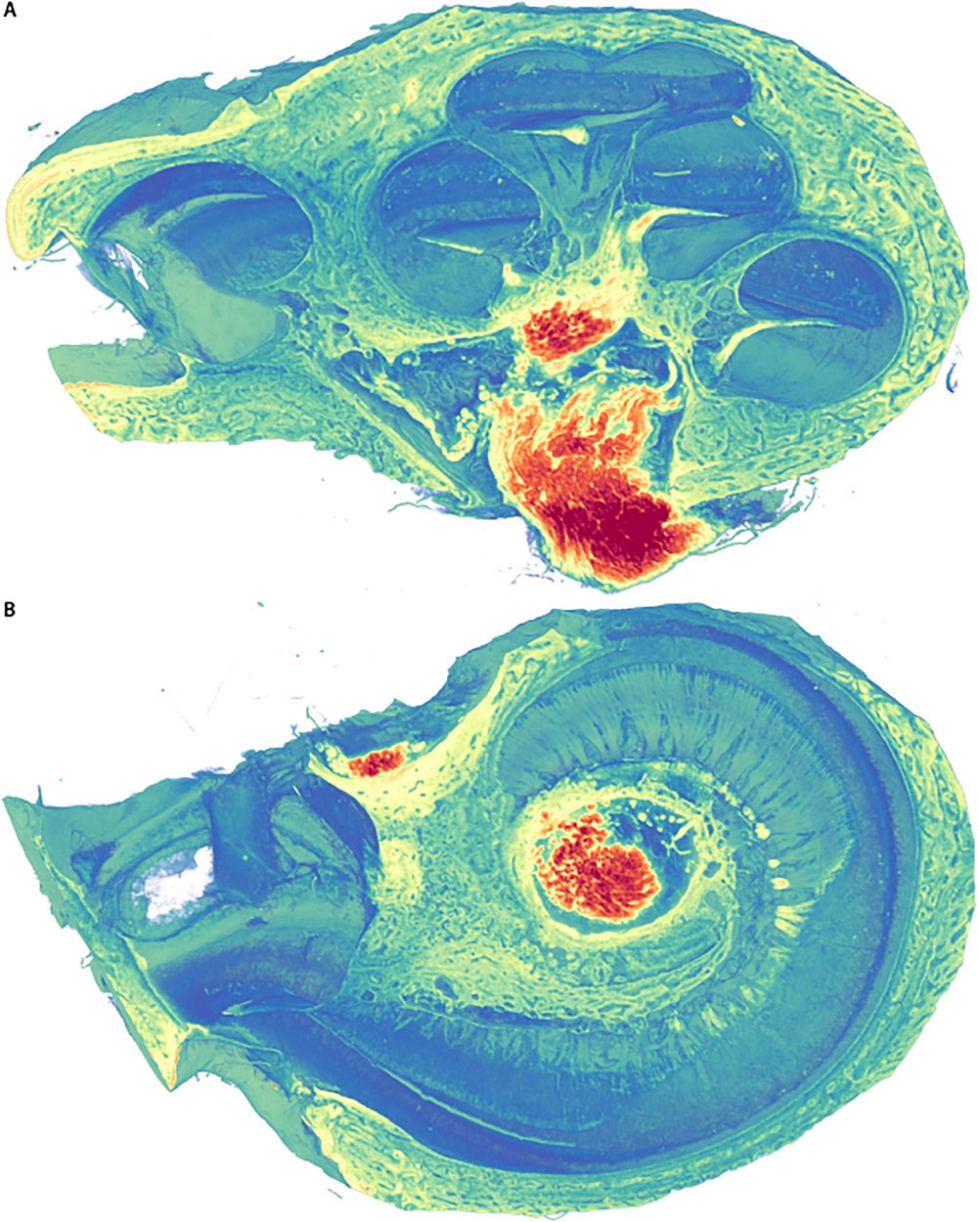
Volume rendering of the OTO stained human cochlea (sample H02) with NVIDIA IndeX. **B** Perpendicular to (**A**). The OTO stain increases contrast for nervous tissue, here shown in light yellow to red.

Next, we illustrate the effect of the OTO staining on XPCT of the cochlea. An overview scan was performed with dx_eff_ = 6.2 μm at an effective energy *E*_eff_ = 94 keV. Subsequently, a ROI tomography with dx_eff_ = 2.3 μm was recorded at *E*_eff_ = 75 keV. The *μ*-CT scan was performed at dx_eff_ = 7.2 μm and a tube voltage of 80 kV. Note that all datasets were registered manually because the sample had to be remounted between all measurements. Of each dataset a slice through the reconstructed volume and a zoom-in is shown in Fig. 7. The overall morphology of the cochlea is represented well in each dataset with the three scalae and separating membranes. The OTO stain increases contrast for lipid-rich tissue components such as the myelin sheaths in nervous tissue. Thus, the central nerve trunk in the modiolus and the innervation to the organ of Corti are highlighted. The *μ*-CT dataset has fairly good quality, while the SR-datasets have a better resolution and better SNR. The SR-dataset with the smallest voxelsize shows even more detail and has a higher SNR. In (B, D) Reissner’s membrane is observed to be ruptured, possibly either due to radiation damage or due to sample handling, i. e. due to bubble formation during degassing or simply remounting of the specimen in the becher. Out of these possibilities, mechanical damage by gas bubble formation seems most likely. Note the sample was first measured with the *μ*-CT, at which point Reissner’s membrane was still intact, and was then degassed again for prolonged time before the synchrotron recording. Volume renderings of the SR overview scan are shown in Fig. 8.

### Decalcified human cochlea

To enhance contrast for soft tissue and to resolve the membranes and the organ of Corti in the cochlea, we have also imaged a decalcified human cochlea (H01) with XPCT. The human cochlea was removed during autopsy and fixed in 4% PFA for several days. After removal of the surrounding bone, the cochlea was decalcified in 10% EDTA for several weeks. The decalcified cochlea was dehydrated in an increasing Ethanol series up to 70% EtOH.

For a polychromatic high-energy beamline like BM18, imaging a weakly absorbing and rather small sample poses a challenge in terms of not oversaturating the detector and not exceeding the X-ray dose on the sample. Furthermore, we reduced the photon energy to have potentially more contrast for soft tissue. The planned installation of a chopper at BM18 which was not available yet during our beamtime would certainly help to solve this problem. We recorded the decalcified cochlea in the horizontalt tail of the BM18 beam with an effective energy *E*_eff_ = 64 keV and a voxelsize dx_eff_ = 1.8 μm. In order to pave a link to higher resolution, we have also scanned this decalcified cochlea during a beamtime at the GINIX instrument^33^, beamline P10, PETRA III at DESY in Hamburg. The GINIX instrument was operated in a parallel beam configuration without collimating optics as described in detail in Frohn et al.^34^. The Energy was set to *E* = 20 keV with a channelcut monochromator. The tomogram was recorded in a continuous rotation at dx_eff_ = 650 nm with a FOV of 1 mm × 1.5 mm. Phase retrieval was performed with a CTF-based approach^35,36^. With an exposure time of 35 ms, a single tomogram is recorded in 2 min and larger volumes can be covered by stitching, see Tab. 3 for a summary of the acquisition parameters.

**Fig. 9:**
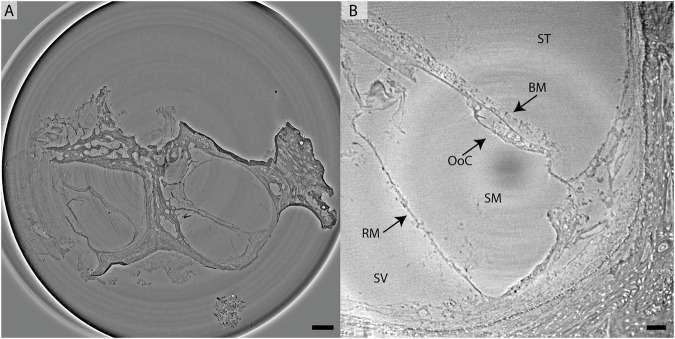
Imaging the decalcified human cochlea in 70% EtOH with XPCT. **A** Virtual slice through the reconstruction volume acquired at BM18 with dx_eff_ = 2.26 μm. Scalebar 500 μm. **B** Virtual slice trough reconstruction volume acquired at GINIX with dx_eff_ = 0.65 μm. The three scalae scala vestibuli (SV), scala media (SM) and scala tympani (ST) are separated by the Reissner’s membrane (RM) and the basilar membrane (BM) with the Organ of Corti (OoC). Scalebar 50 μm.

Virtual slices through the reconstruction volume of both scans are shown in Fig. 9. Filtering in Fourier space was applied to both datasets in order to reduce intensity fluctuations and stripes. This BM18 dataset shows more detail than the prior ones of non-decalcified samples. The soft tissue such as the membranes between the cochlear ducts are represented well. In the GINIX dataset with even higher resolution even cellular structures such as the organ of Corti are recognizable.

## Discussion

We have imaged human temporal bones and cochleae with SR and laboratory *μ*-CT, at different size and preparation of samples, and correspondingly different instrumental settings. Accordingly, the image formation involved different contributions of absorption- and phase-contrast. The phase shift was accounted by a Fourier filter based phase retrieval with adapted parameters based on the Paganin approach derived for a homogeneous object and monochromatic radiation. Despite the fact that strictly speaking these conditions are not fulfilled, the resulting image quality was exceptionally good. The choice of spectra, the instrumental settings and parameters, and the detection technology were all instrumental for the unprecedented image quality obtained. To this end, the tabulated parameters, sample preparation and imaging protocol can serve as a reference for future extension and use, in particular of studies of human temporal bone and cochlea, e. g. for post-mortem analysis of cochlear pathology or implant development.

The high contrast for mineralized tissue can aid in recent studies such as attempts to quantify the bone density in patients with otosclerosis^37^, and 3D analysis of the neo-ossification of the temporal bone in implant patients^38^.

Figure 5 in particular shows that optimized *μ*-CT instrumentation which is more accessible for a preclinical setting will be able to address 3D imaging needs already with a surprisingly high image quality. In this sense, the SR results which still represent an unrivaled benchmark, can be partially translated to compact laboratory scale. At the same time, SR data can provide ground truth for machine learning approaches in image reconstruction and image processing of 3D tomographic data. BM18 as the state-of-the-art SR imaging beamline for whole organ imaging provides data with significantly enhanced visibility for fine details of membranes and the organ of Corti. At the same time, the soft tissue within the cochlea is still too poorly represented due to limited phase-contrast when surrounded by the mineralized and more absorbing components of the temporal bone. Higher image quality for soft nervous tissue therefore requires heavy metal staining, at least when imaged with the necessarily high photon energies to penetrate the temporal bone. To this end, the OTO stain and cochlea preparation which we have already introduced in^11^ comes out at surprising image quality. Even without segmentation, the different contrast values allow for renderings which already represent the structure of the auditory nerve, at least to some extent. At the same time, we have in this work not yet fully exploited the potential of preparing the entire human cochlea by decalcification and selective OTO staining in the organ of Corti, or even decalcified and unstained temporal bones in 70% ethanol.

Higher phase-contrast for unstained soft tissue can be achieved by lowering the photon energy, which becomes possible for decalcified temporal bone. Note as well that for a polychromatic high-energy beamline like BM18, imaging a weakly absorbing and rather small sample can pose a challenge in terms of not oversaturating the detector and not exceeding the X-ray dose on the sample. This is accentuated when reducing the photon energy to increase the contrast for soft tissue. The planned installation of a chopper at BM18 which was not available yet during our beamtime would solve this problem, since spectral tailoring and control of primary beam intensity would then be decoupled. In this way, it should become possible to collect data on temporal bone and cochlea with voxel size of 1 μm to 2 μm in phase contrast mode, with potentially huge biological impact, e.g., in view of localizing and counting hair cells, spiral ganglion neurons, and studies of their innervation. While there is in principle no physical obstacle aside from the necessary implementation of instrumentation, this has not yet been achieved in this work. At the same time, we stress that also at the current setting interesting biology of temporal bone becomes possible at the whole organ level, for example quantification of the bone density in patients with otosclerosis, or 3D analysis of the neo-ossification of the temporal bone in implant patients.

In conclusion, combined and synergistic use of laboratory *μ*-CT instrumentation at optimized settings and dedicated whole organ SR beamlines with large FOVs such as BM18 opens up 3D imaging of human temporal bone for a wide range of biomedical research projects. It also provides data for teaching otorhinolaryngology, for 3D simulation of micromechanics of middle and inner ear, for research addressing hearing disorders and loss, and for implant development. Along with the imaging protocols of this work, all 3D data as well as the raw data is made publicly available as benchmark to compare image quality, as test data to develop further 3D image processing tools, possibly including machine learning, and finally, above all, for future use for biomedical research on the human inner ear. Most interestingly is the fact, that the presented approach and its future extension can bridge histological and micro-anatomical length scales. The 3D XPCT volume data is available at GRO.data (dataset DOI 10.25625/V3YSYD)^39^.

## Methods

### Sample preparation

Temporal bones and cochleae studied here have been collected at the University Medical Center Göttingen (UMG), under proposals #22/1/19 and # 2/8/19 (“analysis of (neuro)degeneration in the cochlea”), approved by the ethics committee of UMG, as well as under #2020P000508 (“Otopathology of the Human Temporal Bone”), approved by the institutional review board (IRB) of the Massachusetts Eye and Ear Infirmary, including appropriate pre-mortem human consent forms and all subsequent procedures for technical processing and HIPAA-compliant data storage. Specimen have been immersed in 4% PFA or PBS without staining except for one cochlea which was OTO stained. For a better overview the specimen are numbered and summarized in Table 1, and the preparation protocols are illustrated in Fig. 1. The cochlea sample H02 was removed at autopsy 14 h post mortem, immersion fixed in 4% PFA for several days and then stained with osmium tetroxide (OTO, OsO_4_), by immersing in 10 ml of 2% OTO in deionized H_20_ at room temperature for 48 h. Round and oval windows were opened and the osmium solution was flushed through the scalae 3 times per day by applying gentle negative pressure near each window. The OTO binds to lipids and is a staining method widely used in electron microscopy^40^. The temporal bone samples H01, H03-H11 have been retrieved at routine autopsy and immersion fixed in 4% PFA for several days. In one of the temporal bone samples (H11) we inserted an optical cochlea implant via the round window, see also^3^. We used an oCI prototype designed for human application which contained 49 micro-LEDs on a strip. The insertion procedure was identical to that of a conventional cochlea implant: After opening of the mastoid cortical bone, a classic mastoidectomy was performed. After that, the round window was exposed by using a posterior tympanotomy approach. The bone covering the round window was removed and thus the round window membrane could be identified and opened. The oCI was then fully inserted into the scala tympani and fixed in position with surgical cement. All samples have been fixed with agarose in cylindrical jars of suitable diameters according to the so-called *HiP-CT protocol*^24^. Due to movement during transport and different requirements to the sample holder, the samples needed to be remounted in new plastic cups between measurements with the inhouse *μ*-CT and the BM18 beamtime.

### Beamline BM18, EBS, ESRF

The source of the BM18 beamline^28^ is a tripole wiggler with a fixed gap. In the central pole (1.56 T), there is higher energy than in the side poles (0.85 T) and this can be exploited to tune the beam intensity and lateral profile on the sample either to measure at a lower intensity or to accommodate for absorption gradients in the sample during off-axis tomography. The polychromatic energy can be tuned between 50 and 280 keV by polished attenuators (i. a. C, SiO2, Al2O3, Al, Ti, Cu, Mo, Ag, W, Au) of different thicknesses. Taking into account the transmission of the entire optical pathway including the sample and the scintillator an effective energy *E*_eff_ can be calculated which corresponds roughly to a monochromatic energy. The 350 mm wide beam at the sample position and different detector configurations allow voxel sizes in the range of 0.7 μm to 80 μm and sample sizes up to 50 cm vertically by 30 cm in diameter for 30 kg maximum. The future large sample stage should make possible to accommodate samples of 2.5 m vertically by 1.4 m in diameter for 300 kg maximum. For image acquisition we used the PCO edge 4.2 with pixelsize dx = 6.5 μm equipped with a threefold Hasselblad tandem optics (100 mm/300 mm) and a 200 μm LuAG:Ce scintillator. Further we used the IRIS 15 detector with dx = 4.25 μm equipped with a zoom optic or demagnifying zoom (dzoom) optic and 0.1 mm or 2 mm LuAG:Ce scintillators respectively. The tomographic scans have been either performed with the rotation axis placed in the middle of the detector or with the rotation axis shifted to one side of the detector (so-called *half acquisition*). Half acquisition is used when the sample is larger than the effective detector width and allows almost twice the FOV in a 360° scan.

The empty beam correction has been carried out either in air for small samples or in the sample environment (jar and solvent) without the sample (*so-called Hierarchical Phase-Contrast Tomography or HiP-CT*^25^). Image reconstruction was performed using *PyHST2* (ESRF), using the Paganin algorithm^32^ for phase retrieval and performing a subsequent tomographic reconstruction with the filtered backprojection. Parameters for each scan are summarized in Tables 2 and 3.

### In-house *μ*-CT

Tomographic scans were acquired using a *μ*-CT instrument (EasyTOM, RX Solutions) equipped with a microfocus source (Hamamatsu L12161-07, W Target, 5–50 μm spotsize) and a flat panel detector (1440 px × 1704 px, dx = 127 μm). As above, empty beam correction has been carried out either in air for small samples or in the sample environment for large samples. The tomographic cone beam reconstruction was performed with the software of the instrument. The acquisition parameters and the parameters of the reconstruction volume for the data presented here are tabulated in Table 4.

### Segmentation and rendering

The segmentation with the thresholding and region growing tools as well as the rendering of these segmentations has been performed with *VGStudio Max* (Hexagon AB, Stockholm, Sweden). The renderings and the animation have been created with *Siemens Cinematic Anatomy* (Siemens Healthineers AG, Erlangen, Germany). The renderings of the stained cochlea have been created using the 3D volumetric visualization framework *NVIDIA Index* (NVIDIA Corporation, Santa Clara, CA, USA).

## Supplementary information

Supplementary Information

Supplementary Movie 1

## Data Availability

The 3D volume data will be available upon publication at GRO.data (10.25625/V3YSYD).The raw beamtime data will become available 2026 at ESRF (dataset 10.15151/ESRF-ES-1223491086).
