# Supplementary material for: 3D imaging of the human temporal bone by X-ray phase-contrast tomography
Source: Npj Imaging. 2025 May 20;3:21. doi: 10.1038/s44303-025-00086-y (PMC12118726; doi:10.1038/s44303-025-00086-y)
Supplement: Supplementary file 1 — Supplementary Information [file 44303_2025_86_MOESM1_ESM.pdf]

Supplemental material for: 3D imaging of the human temporal bone by X-ray phase-contrast tomography

Jannis J. Schaeper, Paul Tafforeau, Christoph A. Kampshoff, Carolina Thomas, Alexander Meyer, Christine Stadelmann, M. Charles Liberman, Tobias Moser, Tim Salditt*

*Correspondence: tsaldit@gwdg.de

Supplemental

In this supplemental we offer additional information on the image chain and present additional datasets from our XPCT studies on the human temporal bone and excised human cochlea.

Supplemental information on the image chain

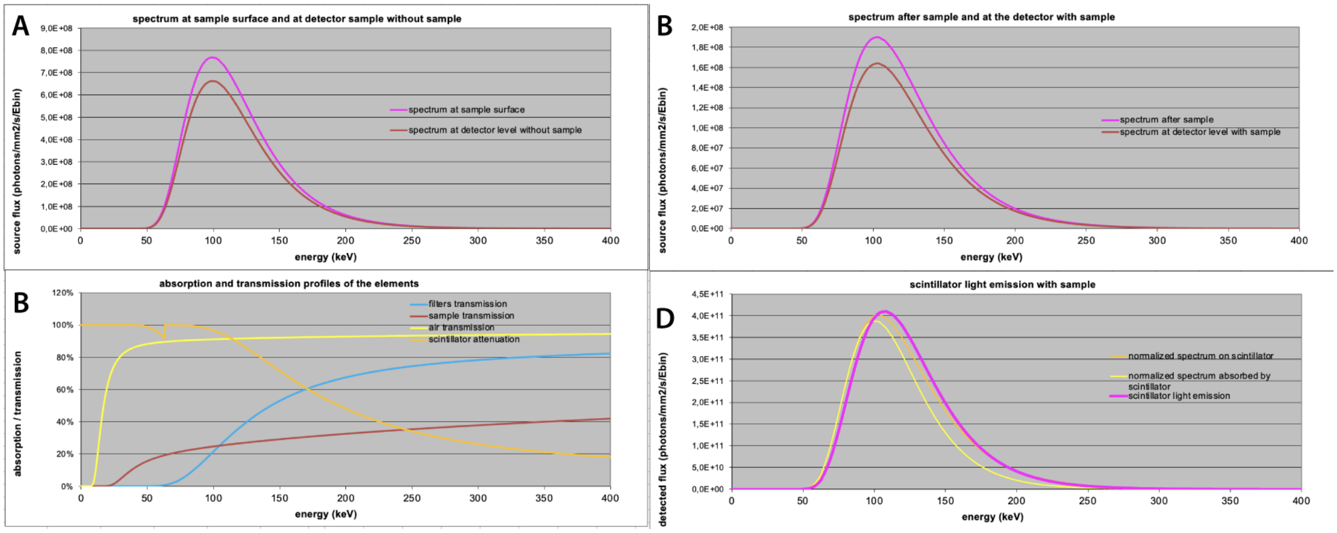

Supplementary Figure 1. Calculated spectra corresponding to an overview scan performed at $E_{\mathrm{eff}} = 125\,\mathrm{keV}$ and $\mathrm{dx}_{\mathrm{eff}} = 6.2\,\mu\mathrm{m}$. (A) Calculated spectrum at sample surface and at detector level without sample. (B) Calculated spectrum after sample and at detector level with sample. (C) Absorption and transmission profiles of filters, sample, air and scintillator. (D) Scintillator light emission with sample.

Human temporal bone

Next, we show another example of the multi-scale imaging capability of BM18 for a human temporal bone immersed in 70 % EtOH. The overview scan was performed with $\mathrm{dx}_{\mathrm{eff}} = 6.2\,\mu\mathrm{m}$ as before. Subsequently a ROI tomography at the position of the cochlea was done with $\mathrm{dx}_{\mathrm{eff}} = 1.8\,\mu\mathrm{m}$. Virtual slices through the reconstruction volume are shown in Fig. 2. The bony structures in the cochlea are represented well and a high detail level in the surrounding bone is visible. The corresponding scan parameters are summarized in the main manuscript.

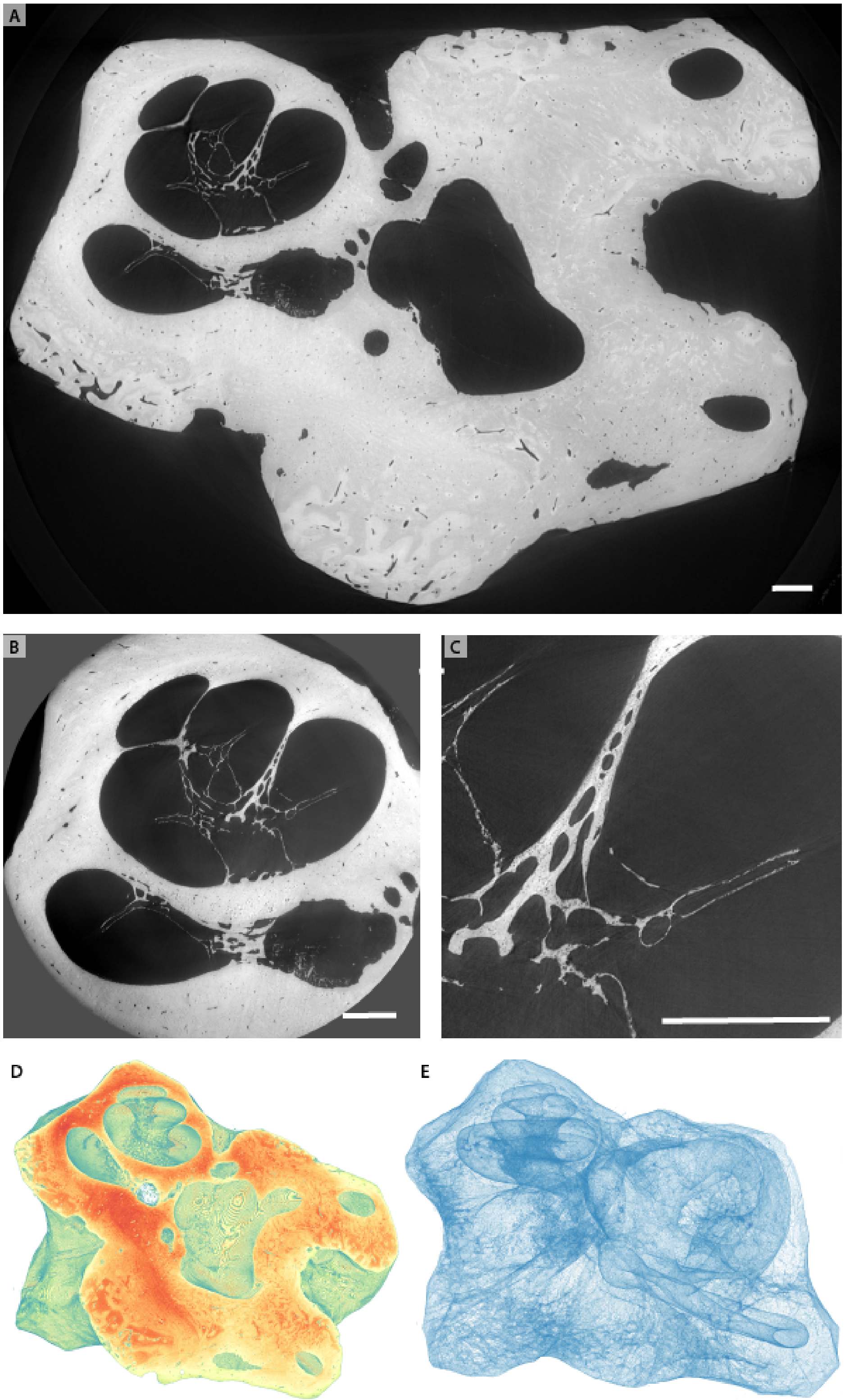

Supplementary Figure 2. XPCT of human temporal bone in 70 % EtOH at BM18. (A) Virtual slice through reconstruction volume acquired at $dx_{eff} = 6.816\,\mu m$. (B) ROI tomography at the position of the cochlea with $dx_{eff} = 1.816\,\mu m$. (C) Zoom into (B). Scalebars 1 mm. (D-E) Volume renderings of the overview scan presented in (A).

OTO-stained cochlea

Finally, we present a second rendering of the specimen H02, in Supplemental Figure 3 the dataset with the higher effective pixelsize of 2.3 µm was rendered.

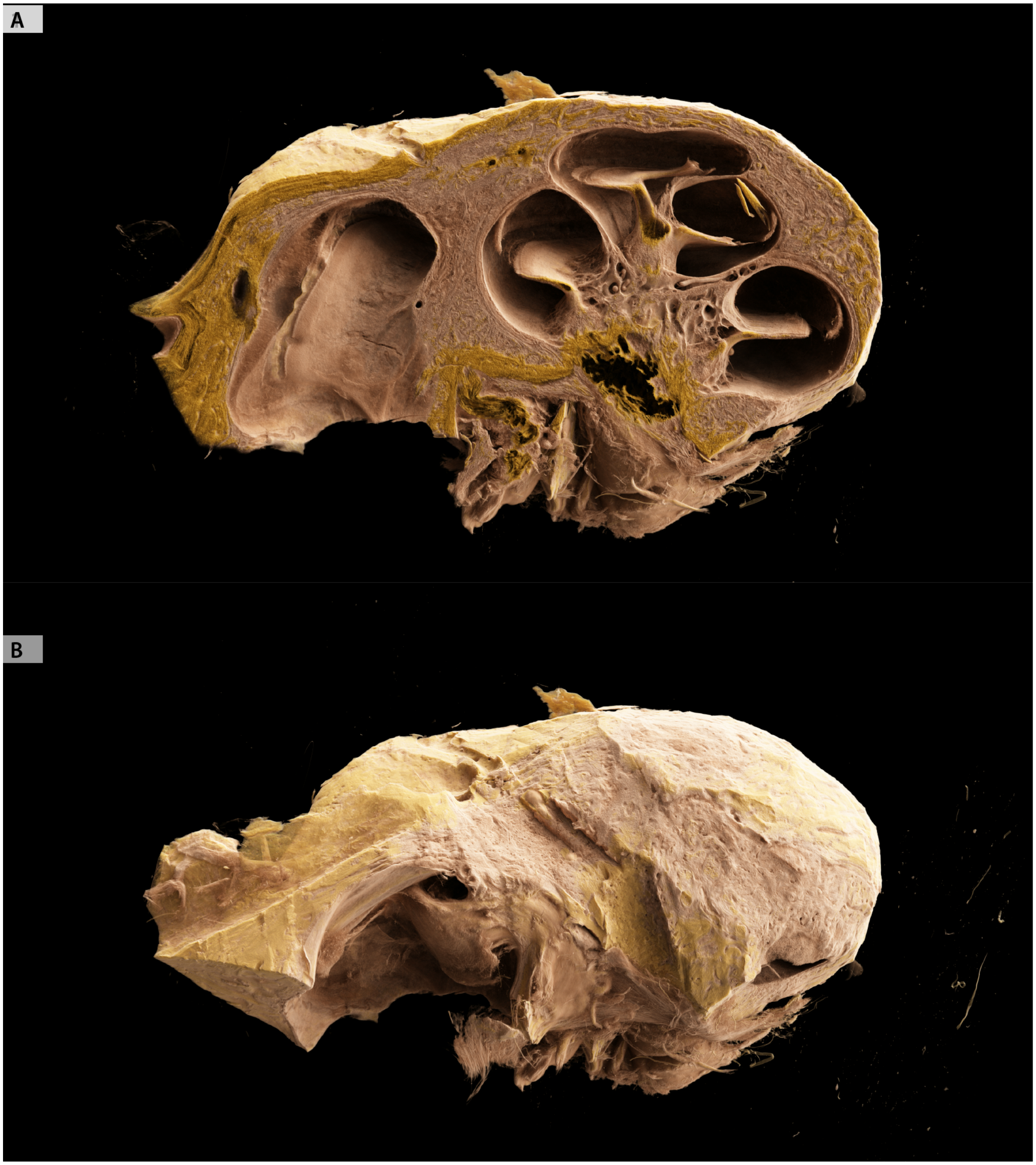

Supplementary Figure 3. Rendering of the OTO-stained human cochlea (H02), $dx_{\mathrm{eff}} = 2.3\,\mu\mathrm{m}$. (A) Half cut through cochlea and (B) complete cochlea (same orientation as in (A)).

Human temporal bone with oCI

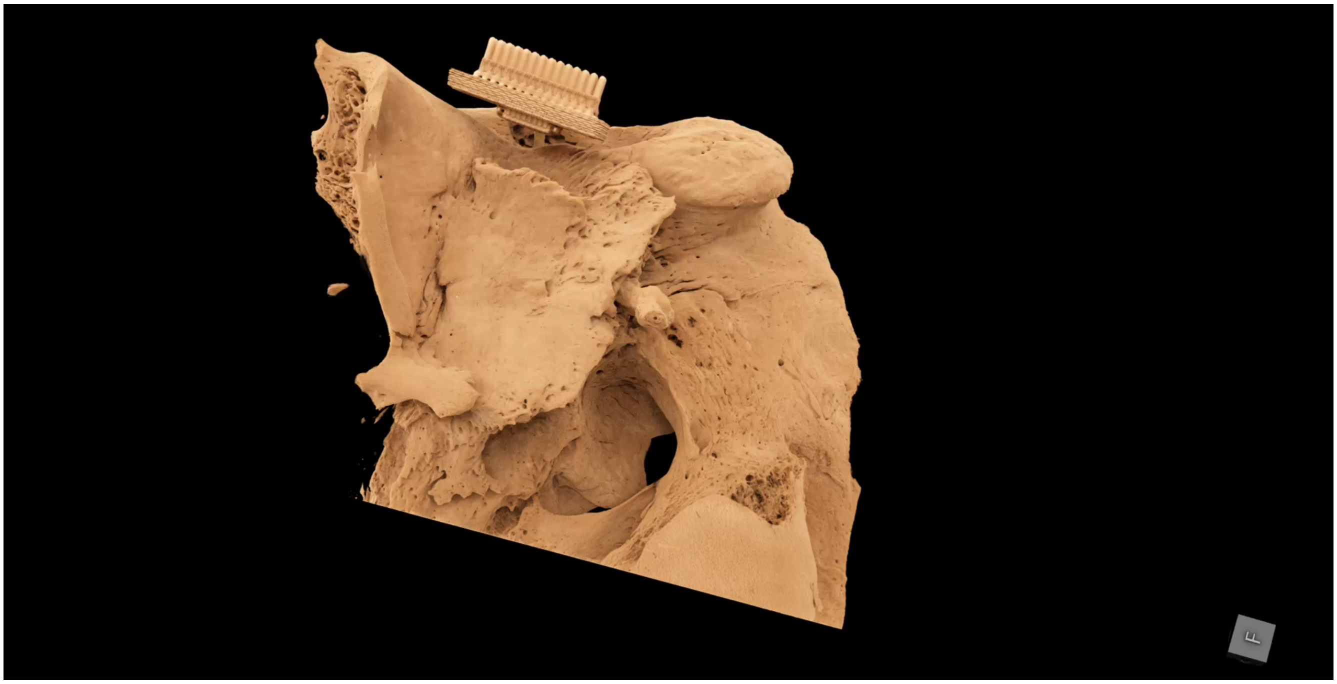

Supplementary Movie 1. The supplementary movie shows an animation of the dataset of the human temporal bone which had been implanted with an oCI post-mortem. The dataset was recorded at the beamline BM18 with an effective pixelsize of 6.2 µm and was presented in Figure 5 and Figure 6 of the main manuscript.
